# Pedicle screw accuracy assessment in ExcelsiusGPS® robotic spine surgery: evaluation of deviation from pre-planned trajectory

**DOI:** 10.1186/s41016-018-0131-x

**Published:** 2018-09-03

**Authors:** Bowen Jiang, A. Karim Ahmed, Corinna C. Zygourakis, Samuel Kalb, Alex M. Zhu, Jakub Godzik, Camilo A. Molina, Ari M. Blitz, Ali Bydon, Neil Crawford, Nicholas Theodore

**Affiliations:** 1 Department of Neurosurgery, The Johns Hopkins School of Medicine, The Johns Hopkins Hospital, 600 N. Wolfe Street, Meyer 7-113, Baltimore, MD 21287 USA; 2 Department of Neurosurgery, Barrow Neurological Institute, St. Joseph’s Hospital and Medical Center, Phoenix, AZ USA; 3 Department of Neuroradiology, Johns Hopkins School of Medicine, The Johns Hopkins Hospital, 600 N Wolfe St, Baltimore, MD 21287 USA; 4 Department of Neurosurgery, Johns Hopkins University School of Medicine, 1800 Orleans Street, Zayed Tower Mailstop 6007, Baltimore, 21287 MD USA; 5 Globus Medical Inc, 2560 General Armistead Ave, Audubon, 19403 PA USA

**Keywords:** Robotic spine surgery, Robot-assisted surgery, ExcelsiusGPS®, Gertzbein-Robbins, Pedicle screw accuracy

## Abstract

**Background:**

The ExcelsiusGPS® (Globus Medical, Inc., Audubon, PA) is a next-generation spine surgery robotic system recently approved for use in the United States. The objective of the current study is to assess pedicle screw accuracy and clinical outcomes among two of the first operative cases utilizing the ExcelsiusGPS® robotic system and describe a novel metric to quantify screw deviation.

**Methods:**

Two patients who underwent lumbar fusion at a single institution with the ExcelsiusGPS® surgical robot were included. Pre-operative trajectory planning was performed from an intra-operative CT scan using the O-arm (Medtronic, Inc., Minneapolis, MN). After robotic-assisted screw implantation, a post-operative CT scan was obtained to confirm ideal screw placement and accuracy with the planned trajectory. A novel pedicle screw accuracy algorithm was devised to measure screw tip/tail deviation distance and angular offset on axial and sagittal planes. Screw accuracy was concurrently determined by a blinded neuroradiologist using the traditional Gertzbein-Robbins method. Clinical variables such as symptomatology, operative data, and post-operative follow-up were also collected.

**Results:**

Eight pedicle screws were placed in two L4-L5 fusion cases. Mean screw tip deviation was 2.1 mm (range 0.8–5.2 mm), mean tail deviation was 3.2 mm (range 0.9–5.4 mm), and mean angular offset was 2.4 degrees (range 0.7–3.8 degrees). All eight screws were accurately placed based on the Gertzbein-Robbins scale (88% Grade A and 12% Grade B). There were no cases of screw revision or new post-operative deficit. Both patients experienced improvement in Frankel grade and Karnofsky Performance Status (KPS) score by 6 weeks post-op.

**Conclusion:**

The ExcelsiusGPS® robot allows for precise execution of an intended pre-planned trajectory and accurate screw placement in the first patients to undergo robotic-assisted fusion with this technology.

## Background

Robotics in spinal surgery have become increasingly prevalent and sophisticated over the last two decades [1–3]. Spinal surgery robots offer several advantages to conventional fluoroscopy-guided or free-hand techniques. In addition to improving screw accuracy, these robots can assist with the development of minimally invasive surgical options and reduce radiation exposure [4, 5]. Institutional experiences have been widely reported with the Spine Assist® (Mazor Robotics, Inc., Caesarea, Israel), Mazor Renaissance™ and the ROSA® (Medtech, Montpellier, France) [6–10]. Recently, a next generation spinal robotic system named the ExcelsiusGPS® (Globus Medical, Inc., Audubon, PA) was approved by the United States Food and Drug Administration (FDA). The ExcelsiusGPS® addresses many limitations of the previous robotic systems, namely the issues with inaccurate registration, inaccurate navigation (due to previous technology utilizing a table-mounted reference frame), loss of tactile feedback, and increased operative time.

Fiducial and surveillance markers are placed in the posterior superior iliac spine (PSIS), outside the surgical field, and a temporary intraoperative CT (ICT) fixture is secured to the skin for patient registration. Notably, pre-operative x-ray, pre-operative CT, or intra-operative CT scans may be utilized for image-guidance. Screw trajectories are planned on the registered images and the trajectory is guided by the floor-mounted robotic arm. Screws are deployed through the rigid tubular robotic arm, thus avoiding the unwieldy patient-mounted frames and flimsy Kirschner wires (K-wires) that previous systems rely upon.

Pedicle screw insertion accuracy in robotic assisted spinal surgery has been well studied and reported to be superior compared to conventional fluoroscopic-guided or free-hand techniques [11–18]. The majority of these studies assess accuracy based on the Gertzbein-Robbins scale, which evaluates for pedicle/cortical breach based on an idealized and optimized trajectory [12]. The topic of how an actual screw placement compares to a pre-operatively planned trajectory in robotic assisted spinal surgery is not well studied [15].

The goal of this study was two-fold: to describe two of the first spinal surgeries with the ExcelsiusGPS® robotic system; and to propose a novel method of assessing pedicle screw accuracy, in the setting of robotic-assistance, by quantifying screw deviations relative to the pre-operatively planned trajectory.

## Methods

### Study design

The study was a retrospective, institution review board (IRB)-approved review of patients who underwent spinal surgery with the ExcelsiusGPS® (Globus Medical, Inc., Audubon, PA) at our institution from October 2017 to December 2017. Patient variables such as demographics, presenting symptoms, Karnofsky Performance Status (KPS), Frankel grade, and ambulatory status were collected. Operative data such as surgical indication, estimated blood loss (EBL), and length of case were also collected.

### Surgical planning and technique

Patients were positioned prone on a Jackson table, with intraoperative neuromonitoring performed throughout the case. After prepping and draping the patient in the usual sterile fashion, the ExcelsiusGPS® fiducial marker (called the dynamic reference base, DRB) was placed in the right posterior superior iliac spine via a small stab incision. A second surveillance marker was placed in the left PSIS with another stab incision. The ICT was attached to the fiducial marker, in a plane parallel to the floor and just above the patient’s skin. The O-arm (Medtronic Sofamor Danek, Inc., Memphis, TN) was then used to obtain a 3-dimensional (3D) CT scan. This was transferred to the ExcelsiusGPS® surgical system for screw planning, which includes entry point, trajectory, screw length, and screw width.

The reference frame ICT was then removed from the field and a typical open spine exposure was performed. For screw placement, the ExcelsiusGPS® robot was draped sterilely, wheeled into the surgical field, and locked securely to the floor. The navigated instruments, including a position tracker, drill, and navigated screw guide were registered with the system. The surgeon utilized a foot pedal to bring the robotic arm to the desired position, corresponding to the screw trajectory planned. With the end effector in position, a power drill was first used to cannulate a trajectory and the screw is then placed through the stable, rigid end effector. Real-time visualization of screw trajectory and indicators of excessive skive force were available to the surgeon.

After instrumentation, laminectomies were performed. Fusion was achieved by using demineralized bone matrix and cancellous autologous bone graft. A post-operative CT scan with 0.5 to 2 mm thickness slices was again performed with the O-arm.

### Screw accuracy assessment

Screw accuracy was assessed using the traditional Gertzbein-Robbins Scale and a newly devised methodology. For the Gertzbein-Robbins method [12], screw accuracy was defined as: perfect intrapedicular localization without any cortical breach (Grade A); < 2 mm pedicle breach (Grade B); < 4 mm pedicle breach (Grade C); < 6 mm pedicle breach (Grade D); ≥ 6 mm pedicle breach (Grade E). Accuracy was assessed by a neuroradiologist (AMB) blinded to the patient’s medical history and treatment, performed on axial, coronal, and sagittal reconstructions from the post-operative 3D CT.

In addition to the Gertzbein-Robbins scale, each implanted screw was evaluated for deviation from a pre-operatively planned trajectory. Deviation was assessed by comparing a 3D offset from the pre-operative plan to the placed screw. Longitudinal length, which does not reflect medial or lateral breach, was not considered for the final analysis. Assessments were performed using dedicated ExcelsiusGPS® software by an engineer (NC) blinded to the patient’s clinical information. Deviation was defined in three data points: distance of placed screw tip from intended trajectory, distance of placed screw tail from intended trajectory, and angular offset.

## Results

### Screw accuracy

Screw accuracy was first graded using the Gertzbein-Robbins method [12], performed by a blinded neuroradiologist. As stated in the literature, clinically acceptable screws are those graded A or B [10, 11]. 100% (8/8) screws were in the clinically acceptable range, with 7 screws deemed Grade A and 1 screw deemed Grade B (less than a 2 mm deviation from the cortex). No screws required revision (Table 1).

**Table 1:** Gertzbein-Robbin assessment of pedicle screw placement

Number of Screws Instrumented (N)	Screw Violation	Gertzbein Robbins Grade
Case 1		
*N* = 4	4/4 screws without violation	4/4 screws Grade A
Case 2		
*N* = 4	3/4 screws without violation	3/4 screws Grade A
	1/4 screw with lateral cortical violation	1/4 screw Grade B

**Fig. 1:**
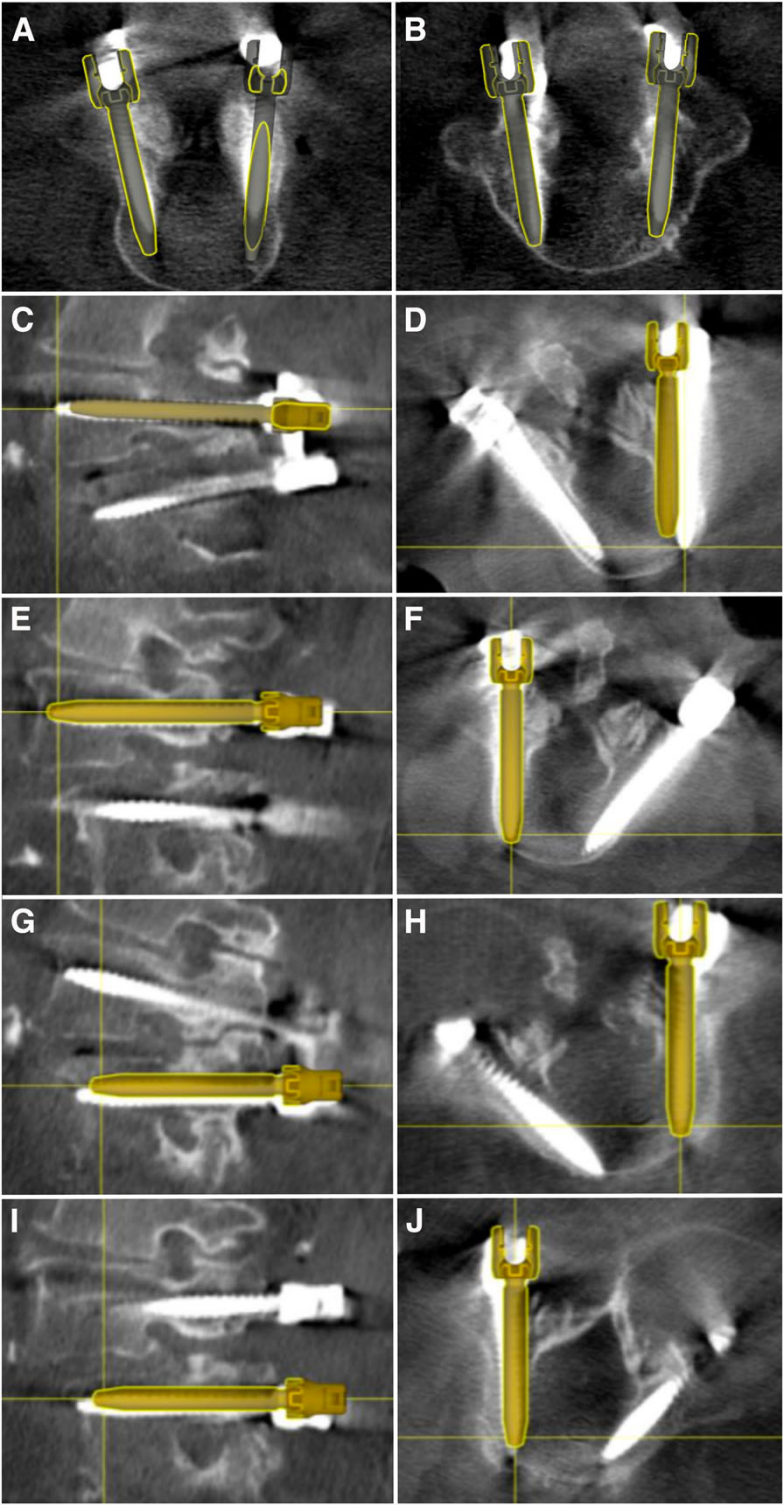
Planned versus placed screw trajectories in ExcelsiusGPS® assisted lumbar fusion. **a** Axial CT overlay reconstruction with placed screw and pre-planned 3D trajectory in Patient 1, who underwent L4 and (**b**) L5 instrumentation. **c-d** Sagittal and axial CT overlay reconstruction in Patient 2 demonstrating deviation of actual right L4 screw placement from pre-planned trajectory, but without medial or lateral cortical breach. **e-f** Left L4 screw placement with mild deviation from pre-planned trajectory on axial and sagittal projections. **g-j** Left and right L5 screw placement in Patient 2 demonstrating minimal deviation from planned trajectory

Deviation of actual screw placement with pre-planned trajectory was also assessed. In all cases, longitudinal screw offset was removed from the final analysis, since screw length is not a predictor of medial/lateral breach. In the first case, the deviated tip distance was < 2 mm in all four screws. In the second case, one screw (left L4) had a > 5 mm deviated tip and tail distance, likely secondary to an entry point that was equivalently offset. Overall, mean screw tip deviation was 2.1 mm (range 0.8–5.2 mm), mean tail deviation was 3.2 mm (range 0.9–5.4 mm), and mean angular offset was 2.4 degrees (range 0.7–3.8 degrees).(Fig. 1, Table 2).

**Table 2:** Deviation assessment of robotic-assisted screw placement compared to pre-operative planned trajectory

	Tip distance (mm)	Tail distance (mm)	Angular offset (degrees)
Case 1			
Left L4	0.9	2.4	2.3
Right L4	1.9	3.1	3.8
Left L5	0.8	3.1	2.9
Right L5	1.6	3.8	3.0
Case 2			
Left L4	5.2	5.4	1.8
Right L4	1.5	0.9	0.7
Left L5	2.9	2.9	1.8
Right L5	2.1	3.8	2.8
Average	2.1 mm	3.2 mm	2.4 degrees

### Demographics and clinical outcomes

**Fig. 2:**
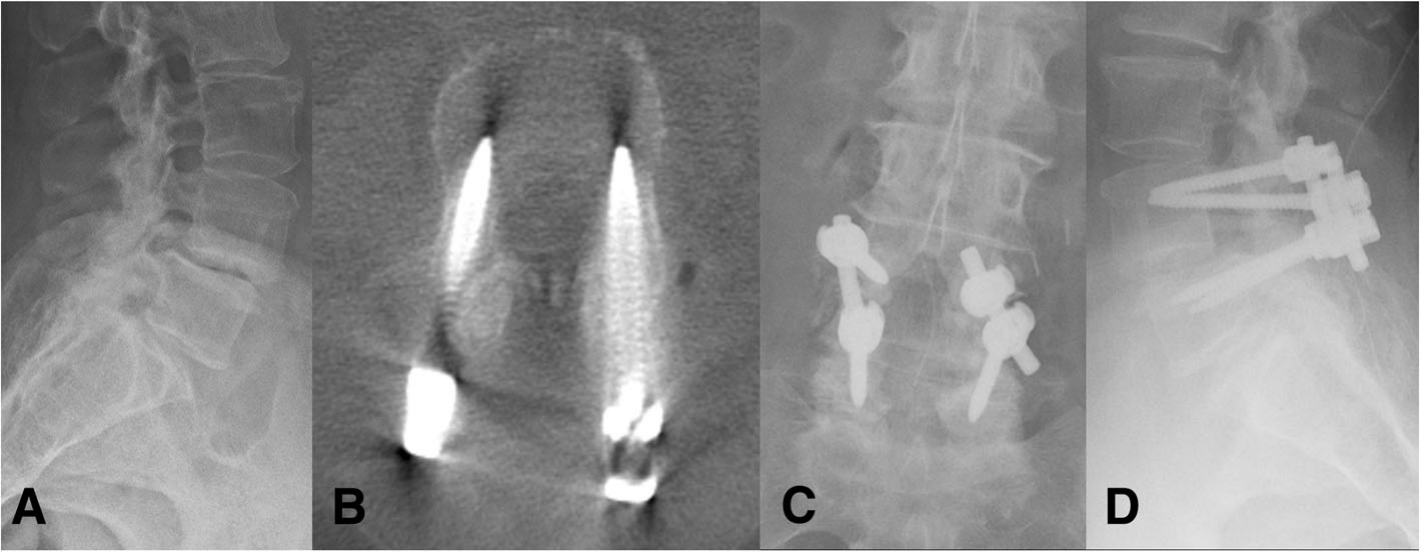
Representative case of a female who presented with radicular pain, weakness, and mechanical back pain due to a degenerative spondylolisthesis at L4/L5. **a** Lateral x-ray demonstrating degenerative spondylolisthesis at L4/L5. **b** Axial post-operative CT with ideal transpedicular screw placement. **c** Post-operative AP x-ray of L4/L5 instrumented arthrodesis. **d** Post-operative lateral x-ray

Two patients were included in this study. Both patients were female and had BMI above 30 (38 and 33, respectively). Both patients presented with radicular leg pain with accompanying mechanical back pain, pre-operative difficulty with ambulation and neurologic deficits, including lower extremity weakness and bladder dysfunction. Both patients were Frankel grade D and had a KPS of 60 at baseline.(Table 3) In both cases, the surgical indication was unstable spondylolisthesis and thus both patients underwent L4–5 laminectomy and L4–5 posterolateral instrumented fusion.(Fig. 2) The mean operative time was 305 min and the average EBL was 250 ml. Both patients experienced improvement in pain at the last follow-up visit. Frankel E grade was noted in both patients and the post-operative KPS was notably improved compared to baseline.(Table 4).

**Table 3:** Clinical presentation and demographics

	Age/ Gender	BMI	Symptoms	Frankel Grade	KPS	Surgical Indication	Operation	EBL	OR time
Case 1	69F	38	LLE pain, weakness, bladder incontinence	D	60	L4–5 unstable spondylolisthesis	L4–5 laminectomy, L4–5 posterolateral fusion	400 ml	403 mins
Case 2	76F	33	RLE pain, weakness, bladder incontinence	D	60	L4–5 unstable spondylolisthesis	L4–5 laminectomy, L4–5 posterolateral fusion	100 ml	207 mins

**Table 4:** Clinical outcomes at last follow-up

	Pain	Neurological Exam	Frankel Grade	KPS	Ambulatory Status
Case 1	Complete resolution	Intact	E	100	Fully ambulatory
Case 2	Improved	Intact	E	70	With walker

## Discussion

Spine surgery robots are becoming increasingly accepted for their various advantages over conventional techniques. For instance, in one study of 112 patients and 536 pedicle screws, the use of the SpineAssist™ surgical robot significantly increased screw accuracy (94.5% vs 91.4%) and decreased radiation exposure (34 s vs 77 s) compared to conventional 2-D fluoroscopy [13]. Our current study corroborate the literature that spinal robots, especially the new ExcelsiusGPS®, improve the accuracy of pedicle screw placement and are clinically safe in patients undergoing instrumented fusion [5, 10, 11]. Furthermore, our proposed screw reproducibility assessment demonstrates that the ExcelsiusGPS® can be utilized for precise execution of an intended, pre-planned trajectory with minimal deviation.

Most literature on pedicle screw accuracy assessment utilize the Gertzbein-Robbins grading scale, which determines acceptability of screw position based on an optimal transpedicular trajectory without cortical violation [12]. Other CT-based grading scales include the Wiesner [19], Rampersaud [20], and Neo systems [21], all of which compare actual screw placement to a universally idealized transpedicular target and consider cortical breach to be suboptimal. However, a purely transpedicular trajectory may not be the best approach in all patients, particularly those with osteoporosis, osteolytic bone, thin pedicles, or oncologic processes. Notably, this novel metric may be applied to various types of spinal screws, other than just pedicle screws, placed with robotic-assistance—not possible with previously described accuracy scales [12, 19–21]. These may include: occipital keel screws, transarticular screws, translaminar screws, C2 pars screws, lateral mass screws, anterior odontoid screws, cortical screws, sacral pedicle screws, S2-alar-iliac (S2AI) screws, and iliac screws. The current grading scales are deficient in that they do not account for deviations from an intended, pre-planned trajectory and do not measure the reproducibility of a specific surgeon directed plan after robotic-assisted instrumentation. Therefore, a more customized accuracy assessment is particularly pertinent in the era of robotic spine surgery.

Van Dijk et al. evaluated for the reproducibility of the surgeon’s plan in robotic- guided percutaneous posterior lumbar interbody fusion using the Mazor SpineAssist robot [15]. The authors fused pre-operative with post-operative CT scans and calculated deviations between planned screw positions with the actual screw positions. Deviation was defined as the perpendicular distance of the midline of the planned screw versus the midline of the actual screw measured in the axial and sagittal planes. The squared root of the summed squared deviation in axial and sagittal planes resulted in the millimeter deviation per screw. Of the 178 screws assessed, mean deviation in entry point was 2.0 ± 1.2 mm and mean difference in angle of insertion was 2.2 ± 1.7 degrees [15]. The authors also reported screw accuracy using the Gertzbein-Robbins scale and found that 97.9% of the screws were Grade A or B.

In another study, Devito et al. performed a similar analysis on planned versus actual placements of 646 screws using the Mazor SpineAssist robot. The authors examined 6 locations on each screw, which defined the entry and exit points from the pedicle and then measured pedicle length in sagittal and axial views to create a 3D positional and orientation accuracy assessment [22]. A mean deviation of 1.2 ± 1.5 mm on the axial plane and 1.1 ± 1.2 mm on the sagittal plane was reported. 98.3% of the screws were Grade A or B on the Gertzbein-Robbins scale [22]. These findings are consistent with our results, which show that ExcelsiusGPS® can achieve plan reproducibility to approximately 2 mm deviation and 2 degree offset with 100% acceptable accuracy on the Gertzbein-Robbins evaluation.

While most studies on this topic draw comparisons between two fused CT scans (pre-op versus post-op), Tsai et al. proposed an intraoperative robotic classification system to assess the accuracy of K-wire placement as a predictor of pedicle screw accuracy [23]. In their study of 176 Renaissance robotic assisted pedicle screw instrumentation, the intraoperative accuracy of K-wire placement was assessed using the Renaissance robotic system itself, which served both diagnostic and instrumentation purposes. The authors defined less than 3 mm deviation of placed K-wire from the pre-planned trajectory as malpositioned and found that 94.9% of K-wires placed were considered acceptable before repositioning. Delayed, post-operative CT was used to validate this intraoperative classification system and there was no statistical difference in screw accuracy between the proposed system and the more conventional pedicle accuracy assessment scales [23].

The ExcelsiusGPS® robotic system has several technological improvements over prior robotic systems in spinal surgery, particularly as it relates to the usage of K-wires. The Mazor SpineAssist and Renaissance systems rely on an interspinous clamp attached to 3D fiducial markers for patient registration. Robotic arms are then screwed onto the interspinous clamp, and a guide tube allows for cannulation and the insertion of K-wires. The interspinous clamp can be disrupted with inadvertent motion and can become malpositioned during drilling. Since the arms are screwed on the interspinous clamp attachment, they are not rigid enough for direct screw insertion and thus a K-wire must first be used [1–3, 14]. The ExcelsiusGPS® system does not rely on an interspinous clamp but rather a fiducial array and surveillance marker placed on the PSIS, away from the working field. A rigid robotic arm with 6 degrees of freedom can withstand significant force without displacement. As such, surgeons can drill and place screws directly through the guide tube of the robotic arm, without the use of K-wires.

Limitations of our study include the small sample size and single institution study. One of the current drawbacks is the significant time required for the two O-arm spins, which were done early in our series to assess accuracy and is no longer routinely done. In addition, only medial/lateral (axial plane) and superior/inferior (sagittal plane) deviations were utilized for our accuracy assessment, since the longitudinal depth of the screw is operator dependent and not necessarily a predictor of screw accuracy.

## Conclusion

The new ExcelsiusGPS® robot has several technological advances over prior spine robots and allows for precise execution of an intended pre-planned trajectory and accurate screw placement in the first patients to undergo robotic-assisted fusion with this technology.

## Abbreviations

DRB: Dynamic reference base
EBL: Estimated blood loss
FDA: Food and Drug Administration
ICT: Intraoperative CT
IRB: Institution review board
KPS: Karnofsky Performance Status
K-wires: Kirschner wires
PSIS: Posterior superior iliac spine
S2AI: S2-alar-iliac
